# CVII: Enhancing Interpretability in Intelligent Sensor Systems via Computer Vision Interpretability Index

**DOI:** 10.3390/s23249893

**Published:** 2023-12-18

**Authors:** Hossein Mohammadi, Krishnaprasad Thirunarayan, Lingwei Chen

**Affiliations:** Department of Computer Science and Engineering, Wright State University, Dayton, OH 45435, USA; mohammadi.5@wright.edu (H.M.); lingwei.chen@wright.edu (L.C.)

**Keywords:** intelligent sensor, deep neural network, interpretability, computer vision

## Abstract

In the realm of intelligent sensor systems, the dependence on Artificial Intelligence (AI) applications has heightened the importance of interpretability. This is particularly critical for opaque models such as Deep Neural Networks (DNN), as understanding their decisions is essential, not only for ethical and regulatory compliance, but also for fostering trust in AI-driven outcomes. This paper introduces the novel concept of a Computer Vision Interpretability Index (CVII). The CVII framework is designed to emulate human cognitive processes, specifically in tasks related to vision. It addresses the intricate challenge of quantifying interpretability, a task that is inherently subjective and varies across domains. The CVII is rigorously evaluated using a range of computer vision models applied to the COCO (Common Objects in Context) dataset, a widely recognized benchmark in the field. The findings established a robust correlation between image interpretability, model selection, and CVII scores. This research makes a substantial contribution to enhancing interpretability for human comprehension, as well as within intelligent sensor applications. By promoting transparency and reliability in AI-driven decision-making, the CVII framework empowers its stakeholders to effectively harness the full potential of AI technologies.

## 1. Introduction

The realm of intelligent sensors has witnessed a surge in the utilization of advanced Machine Learning (ML) models [1,2,3], with applications in domains such as intelligent surveillance, healthcare diagnostics, and autonomous vehicles [4,5,6,7], leading to their enhanced capabilities for predicting a wide variety of phenomena [8]. However, this improved effectiveness has come at the cost of comprehending the inner workings of the decision models [9]. As these tasks were traditionally the purview of human operators, the imperative for transparency in the decision-making processes of ML algorithms has grown significantly. This transparency is essential in building trust and confidence in the outcomes generated by intelligent sensor systems.

Deep Neural Network (DNN) models, a subset of ML models, have particularly flourished in intelligent sensor applications, demonstrating remarkable success in computer vision [10,11], speech recognition [12], natural language processing [13], and numerous other fields [14,15,16]. Nevertheless, these models occasionally exhibit perplexing behaviors. For instance, in computer vision tasks, researchers have uncovered a phenomenon known as “adversarial examples”, where DNNs, while achieving state-of-the-art performance, can be easily manipulated by imperceptible input perturbations, defying human comprehension [17]. These subtleties challenge the reliability and safety of intelligent sensor systems.

In response, researchers have tried to unravel the interpretability of ML models, especially DNNs, to demystify their opaque nature [18]. Machine learning interpretability lacks a universally agreed-upon definition. Some define interpretability as the degree to which a human can understand the cause and effect within a system or a model’s predictions [19]. Ref. [20] emphasized the need for models to provide explanations that are understandable and relevant to human users. In contrast, others have focused on transparency, asserting that an interpretable model should reveal its internal mechanisms in a clear and accessible manner [21,22]. Ref. [23] stressed the importance of understanding both the algorithms and hardware implementations in the context of computer vision. This diversity in definitions highlights the multidimensional nature of interpretability, encompassing various aspects such as understandability, transparency, and relevance. The lack of consensus underscores the complexity of the interpretability concept in machine learning, reflecting the diverse needs and perspectives of stakeholders.

However, the formalization and quantification of interpretability remain elusive, with some likening it to the adage, “you will recognize it when you see it”. This inherent ambiguity necessitates a more structured and systematic approach. Presently, interpretability assessments fall into two main categories: human-centered evaluation, involving domain experts or laypersons; and objective evaluations, offering quantifiable metrics [24]. Interpretability, in this context, denotes the model’s capacity to elucidate its decisions in human-understandable terms, leveraging domain-specific knowledge [25]. These explanations empower humans to validate the predictions made by a ML model. We provide an overview of key interpretability methods as follows, including SHAP (SHapley Additive exPlanations), LIME (Local Interpretable Model-agnostic Explanations), occlusion detection, and saliency maps, which have significantly contributed to enhancing the interpretability of machine learning models:

- **SHAP (SHapley Additive exPlanations):** Originating from cooperative game theory, SHAP values offer a comprehensive understanding of feature importance by quantifying the contribution of each feature to a model’s output [19]. The model-agnostic nature of SHAP makes it adaptable to diverse machine learning models;
- **LIME (Local Interpretable Model-agnostic Explanations):** LIME addresses the challenge of complex global model behavior by creating interpretable surrogate models for local input space regions [20]. It generates perturbed samples, observes model predictions, and constructs interpretable models, providing locally faithful explanations;
- **Occlusion Detection:** This method involves systematically occluding specific input regions, such as portions of an image, to observe changes in model predictions [21]. It proves valuable in image-based models, revealing the importance of different regions in influencing predictions;
- **Saliency Maps:** Particularly prominent in computer vision tasks, saliency maps highlight relevant input regions influencing model decisions [22]. By calculating gradients with respect to input features, these saliency maps visualize areas crucial for the model’s output.

As the existing interpretability solutions tend to be qualitative, they fail to quantify the adequacy of a model’s decisions [26] and some do not reflect the intrinsic cognitive processes underlying a ML model. Our work introduces the Computer Vision Interpretability Index (CVII) to improve quantification, which is applicable to intelligent sensor applications, particularly in computer vision. By employing CVII, end-users can gauge the interpretability level associated with images and models in their applications, thereby making informed decisions on the trustworthiness of the model’s output.

CVII operates by calculating an interpretability index for input images, exploring various facets such as object recognition, classification accuracy, and object separation within images. It encompasses essential aspects like bounding boxes, segmentation annotations, and categories, aligning with standard tools and annotations in the realm of computer vision. To validate our approach, we experimented using benchmark datasets. The evaluation results demonstrated the efficacy of CVII in quantifying the interpretability of DNNs in computer vision tasks. This quantification aids in assessing the reliability and effectiveness of their decision-making processes.

In summary, our contributions are as follows:

- We propose an interpretability index tailored for intelligent sensor applications, focusing on image classification, object detection, and semantic segmentation;
- We introduce a Computer Vision Interpretability Index benchmark that leverages the COCO (Microsoft Common Objects in Context) test set annotation as a reference, enabling developers to assess their models’ contributions to interpretability;
- We provide comprehensive experimental evidence showcasing the consistency and reliability of our CVII approach, both in benchmark comparisons and real-world scenarios.

The remainder of this paper is organized as follows: Section 2 offers an in-depth overview of the primary tasks within intelligent sensor applications with illustrative examples from the domain of computer vision, in two distinct contexts: first, in establishing a benchmark for model evaluation, and second, in a real-world setting, to assess the alignment with the common sense of downstream systems. Section 3 presents a detailed analysis of the experimental results in both settings. Section 4 provides an extensive discussion of the challenges encountered during our research. Finally, Section 5 provides lessons learned and concludes.

## 2. Computer Vision Interpretability Index for Intelligent Sensors

### 2.1. Terminology: Main Tasks in Computer Vision

Image classification, object detection, and semantic segmentation are fundamental tasks in computer vision [23]. When integrated into intelligent sensors, these tasks can enhance the sensor capabilities in various applications, ranging from surveillance to autonomous systems, by providing critical scene analysis and understanding. In this section, we review these tasks and their associated models.

- **Image Classification:** Image classification is the task of assigning a single label or class to an entire image. Models such as ResNet50, DenseNet161, VGG-16, Inception-V3, and EfficientNet are commonly used for image classification [27]. In the realm of intelligent sensors, image classification empowers vision-based sensors to recognize and categorize scenes or objects in real time. For example, a smart traffic camera equipped with image classification capabilities can identify various types of vehicles, facilitating traffic monitoring and management.
- **Object Detection:** Object detection aims to locate objects within an image by providing bounding box coordinates and class labels for each detected object. There are two main categories of methods: one-stage methods and two-stage methods. One-stage methods like YOLO, SSD, and RetinaNet prioritize fast inference, while two-stage methods like Faster R-CNN, Mask R-CNN, and Cascade R-CNN focus on detection accuracy [28]. In the context of intelligent sensors, object detection is crucial for applications such as autonomous identification of pedestrians, vehicles, and obstacles, enabling autonomous navigation and collision avoidance.
- **Image Segmentation:** Image segmentation involves pixel-level semantic labeling to classify pixels (i.e., semantic segmentation) or to identify and delineate specific objects (i.e., instance segmentation) or both (i.e., panoptic segmentation) [29]. In the realm of intelligent sensors in vision, image segmentation is essential for fine-grained scene understanding. In autonomous robotics, sensors with image segmentation can identify objects of interest, enabling precise manipulation and navigation in unstructured environments.
- **Benchmark Dataset:** The Microsoft Common Objects in Context (MS COCO) dataset is widely used for object detection, segmentation, and captioning tasks [30]. It consists of images depicting everyday situations with annotated instances of 91 object categories. In this study, we utilized the COCO dataset, specifically the images and their annotations, to train and evaluate our approach.

### 2.2. Problem Statement and Challenge

In recent years, researchers have made significant strides in elucidating the interpretability of machine learning models applied to intelligent sensor data, striving to develop methodologies for assessing the interpretability of these models’ decisions [31]. However, many of the existing solutions are qualitative and opaque regarding how the models arrive at their final decisions [26].

Users of intelligent sensor systems increasingly demand interpretability, as they rely on sensor-generated insights to make critical decisions. Quantitative perspectives can bolster their confidence in these model outputs and enable effective comparison among alternative models.

To address these challenges, we propose a Computer Vision Interpretability Index (CVII) tailored for intelligent sensors. This index aims to be accessible to both experts and non-experts, offering a quantitative model for machine learning interpretability that can be universally applied across a broad range of intelligent sensor applications.

### 2.3. Overview of CVII

The primary objective of formalizing the degree of interpretability is to capture how humans comprehend related sensor data patterns. Just as neural network architectures draw inspiration from the structure of the human brain, successful intelligent sensor data analysis algorithms often strive to emulate human decision-making processes [32]. We introduce a Computer Vision Interpretability Index (CVII) for intelligent sensors that leverages human-like inference in sensor data analysis, demystifying the inner workings of machine learning models applied to sensor data.

To construct a quantitative model of interpretability, we will focus on three fundamental applications: image classification, object recognition, and semantic segmentation. For instance, in the process of visual comprehension, individuals start by identifying objects, followed by discerning their respective types, determining the relative arrangement of objects, and finally establishing the boundaries between objects and their backgrounds. By incorporating these fundamental steps into our model, we develop a robust framework for interpreting machine learning outcomes in the context of computer vision tasks. The CVII offers two key advantages that enhance our understanding of sensor data/model interpretability, as elaborated below:

- First, for end-users utilizing deep learning models in computer-vision-related sensors, our index enables calculating the interpretability of an image by simply inputting the image into the model. This functionality is critical for ensuring that the outputs generated by machine learning algorithms are reliable and effective for decision-making purposes. By utilizing the CVII, users can gain valuable insights into the factors influencing the model’s predictions and grasp how the algorithm arrived at its conclusions.
- Second, our proposed model serves as a computational framework for comparative evaluation using the CVII. The CVII is calculated by averaging it across a benchmark dataset, which serves as our gold standard. Other developers can compare their models against this gold standard by incorporating their own models as part of our framework. This comparison enables developers to assess how closely their models approach the human-level interpretability provided by the benchmark dataset.

**Figure 1:**
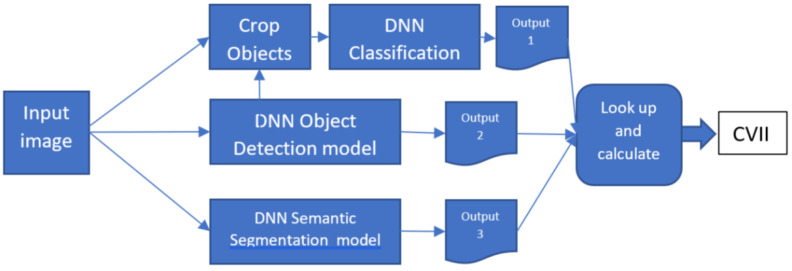
Computer Vision Interpretability Index calculation setup tailored for intelligent sensor data analysis in a real-world setting.

In summary, the first point captures the intrinsic complexity of an image from a computer vision applications perspective, while the second point refers to its value for comparing different computer vision applications. Additionally, establishing a benchmark is crucial for standardization and model evaluation in intelligent sensor data analysis. Many datasets already include annotations such as pattern types, anomaly labels, and maintenance event predictions. The CVII can be appended to the dataset file. However, assessing machine learning model decisions in practice requires an ensemble of models for pattern recognition, anomaly detection, and predictive maintenance during inference. Figure 1 illustrates the structure of our proposed model for CVII calculation.

### 2.4. Interpretability Index Formulation

In the context of intelligent sensors, especially within the domain of smart cameras, the CVII plays a pivotal role in enhancing the capabilities of these sensors by enabling evaluation of machine learning-based models for accurate interpretation of visual data.

Now that the model’s basic structure has been established, we develop the specifics of computing the CVII from various perspectives. *This index is divided into three parts relevant to human assessment: the degree of recognition of image objects, the accuracy in classifying all objects, and the degree of separation of different objects in an image.* We leverage image characteristics such as bounding box parameters, segmentation annotation, and category to calculate the CVII, as these are natural and common in the annotation of image collections, as well as the output of various deep learning algorithms in computer vision. Furthermore, these are conceptually meaningful and human-accessible. The technical details about each of these components are specified below.

**Figure 2:**
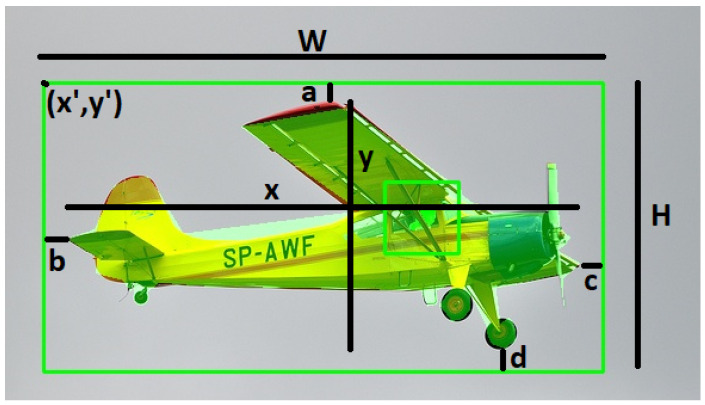
Illustration of annotations used in the CVII (Computer Vision Interpretability Index). This figure provides a visual representation of the annotations used in the CVII framework, clarifying their respective meanings and purposes.

- **Detection Ratio.** The first part of the CVII captures *the recognizability of the objects in an image*, which is defined as
  (1) $$\mathrm{DR}=\frac{\mathrm{OD}}{\mathrm{OE}},$$
  where DR stands for detection ratio, OD stands for Objects Detected, and OE stands for objects exist. In the creation phase with a benchmark, we can collect the OD from the number of objects recognized by the model under construction and the OE from the annotations in the benchmark dataset for the image under examination.
- **Intersection over Union-Anchor Segmentation.** The second part, which captures *the resolution of the objects in the image*, is computed using the three-part formula below:
  (2) $$\mathrm{IoU}\_\mathrm{AS}=\frac{1}{n}\sum_{i=1}^{n}\left({\mathrm{AF}}_{i}+{\mathrm{AC}}_{i}+{\mathrm{ASMM}}_{i}\right)$$
  where IoU_AS represents the intersection over union-anchor segmentation that *assesses the accuracy and fitness of bounding boxes and their annotations for objects in an image*, where the greater the agreement between the two, the more likely the object can be detected. Specifically, for each bounding box *i*, AF, the anchor fitness is computed by summing the distances between the object and anchor box edges (i.e., distances *a*, *b*, *c*, and *d* of the four edges), divided by the object’s bounding box dimensions (i.e., width *W* and height *H*):
  (3) $$\mathrm{AF}=\frac{\left|a|+|b|+|c|+|d\right|}{W+H}$$
  Using the following equation, *the centrality of the anchor box in the image can be determined*. AC stands for anchor centerness, which is calculated as
  (4) $$\mathrm{AC}=\sqrt{{\left(\frac{x}{2}-\left(x^{\prime }-\frac{W}{2}\right)\right)}^{2}+{\left(\frac{y}{2}-\left(y^{\prime }-\frac{H}{2}\right)\right)}^{2}}$$
  ASMM, or anchor segmentation mismatch, is calculated using anchor segmentation unity (ASU), which assesses *the overlap between the anchor and the segmentation*:
  (5) $$\mathrm{ASMM}=\left\{\begin{array}{cc} 0 & \mathrm{if}\;\;\;\mathrm{ASU}\geq 0.5\\ 0.5-\mathrm{ASU} & \mathrm{if}\;\;\;\mathrm{ASU}<0.5 \end{array}\right.$$
  where an overlap of more than 50 percent indicates that the object is less likely to be obscured or divided. ASU is computed by counting the pixels in the segmentation that overlap with the anchor box.
- **Classification Rate.** All relevant metrics above can be derived from the output of computer vision algorithms and annotations in computer vision benchmarks. However, the accuracy of classification is only relevant when the method is applied in practical scenarios. This component is determined using the following formula:
  (6) $$\mathrm{CR}=\sum_{i=1}^{n}{\mathrm{CC}}_{i}*\frac{{\mathrm{AnchorSize}}_{i}}{\mathrm{ImageSize}}$$
  This metric, denoted as CR or classification rate, is a *measure of the accuracy of object classification* in computer vision systems. The underlying factor for CR is the value of classification correctness (CC), which is determined by analyzing the outputs of image classification, object recognition, and semantic segmentation applications. To ensure that CR remains within the range of 0 to 1, we incorporate a ratio term (AnchorSize/ImageSize). The membership of an object to a particular class is used to determine CC. When all three applications recognize the same class, the algorithm’s average confidence percentage is used to calculate CC. If the outputs of the three applications do not agree and assign different classes, CC is the average confidence of the applications that agree on the same class.
- **Computer Vision Interpretability Index (CVII).** Finally, the formula for computing the CVII is defined as follows:
  (7) $$\mathrm{CVII}=\frac{\lambda_{1}\mathrm{DR}+\lambda_{2}\mathrm{CR}-\lambda_{3}\mathrm{IoU}\_\mathrm{AS}}{\lambda_{1}+\lambda_{2}}$$
  where $\lambda_{1}$, $\lambda_{2}$, and $\lambda_{3}$ are integer hyperparameters that can be determined at the time of use, depending on the importance of each of the three components for the computer vision task. Note that IoU_AS has a punitive aspect, because it assumes that the algorithm correctly segments the pixels and determines the bounding boxes so that the rest of the analysis is meaningful, and if they do not match, the CVII of the image is reduced. Figure 2 provides a visual representation of the relevant annotations. Let $x^{\prime }$ and $y^{\prime }$ be the coordinates of the upper-left corner of the bounding box, *W* be the width of the bounding box, *H* be the height of the bounding box, *x* be the real width of the object, and *y* be the real height of the object. Let *a* be the distance between the upper edge of the bounding box and the top of the object, *b* be the distance between the left edge of the bounding box and the left side of the object, *c* be the distance between the right edge of the bounding box and the right side of the object, and *d* be the distance between the lower edge of the bounding box and the bottom of the object.
- **CVII for Intelligent Sensors.** Intelligent sensors equipped with DNNs can leverage the CVII, not only to recognize objects in images, but also provide insights into the factors influencing the model’s predictions. This is essential for ensuring safety, security, and precision in applications. Moreover, the CVII offers a benchmark for evaluating the interpretability of intelligent sensor models. Developers can compare their models against this benchmark, allowing them to assess its accessibility to humans, as provided by benchmark datasets and annotations. This comparison aids in standardizing and improving evaluation, ultimately leading to more reliable and trustworthy systems.

In summary, the CVII bridges the gap between advanced machine learning models, their applicability to intelligent sensors, and human-centric interpretability, enhancing their utility and reliability in various applications, including surveillance, autonomous systems, and industrial automation.

## 3. Experimental Results and Analysis

**Figure 3:**
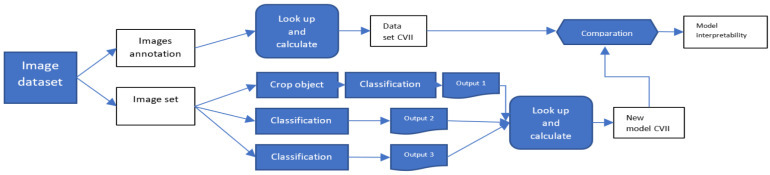
Model interpretability comparison setup. We calculated the overall COCO test set interpretability using the proposed approach and the annotations provided by the dataset (the first row in the diagram). Model developers can substitute their model with one, two, or all three component tasks in the computer vision and calculate the interpretability index based on the annotations their model provides. The results can then be compared with the benchmark provided in the first row.

The proposed CVII serves two primary objectives, and accordingly experiments were conducted in two distinct settings. The first setting involved assessing the index’s performance on the COCO dataset, establishing a benchmark reference for comparing different models’ contributions to interpretability, as illustrated in Figure 3. In the second setting, we present a methodology for using the index to evaluate any image or computer vision model in real-world scenarios. This study aims to provide a comprehensive and effective method for evaluating the interpretability of computer vision models, which has relevance in various computer vision applications, as depicted in Figure 1.

### 3.1. Implementation Details

We developed a Python program to assess the CVII of an image using the process and formulas outlined in the preceding sections. This program takes an image as input and generates annotations for object detection, semantic segmentation, and classification. The resulting annotations are stored in a dictionary with the image name as the key. The dictionary structure is as follows:

		annotation = {’image_name’: name_only,
		’objects_seg’: {}, ’objects_OD’: {},
		’objects_class’: {}, ’CVII’:{}}
		  

To generate these annotations, we detected objects using the YOLOv5 model. The dictionary stores the resulting confidence scores, object types, bounding box coordinates, and image IDs. These bounding box coordinates are then used to crop the objects from the image and input them into our fine-tuned classification model, as explained in the previous section. The outputs of the classification model, including class labels and confidence scores, are added to the same dictionary alongside the corresponding information for each object in the image.

Subsequently, we utilized the Mask R-CNN ResNet50 FPN model for semantic segmentation. The results for each object in the image, such as object type, segmentation score, area, center of object, upper left corner of the segmented object (calculated using output contours), width, and height of the segmented object, are appended to the same dictionary. By following these steps, we acquire all the information necessary to calculate the CVII for an image. We initially compute the CVII for each object and then average these CVIIs to obtain a unique index for the image’s CVII.

This process was executed on all the images in the COCO dataset’s test set. The training set was not utilized, as some of the computer vision models we employed were trained on these images. Our model’s evaluation was performed on the COCO test set, to ensure consistency with real-world performance. The outcomes of this evaluation are discussed below.

### 3.2. CVII for the COCO Dataset

We evaluated the CVII for all images in the COCO test set (4975 images), we aimed to demonstrate the effectiveness of our method in assessing the interpretability of computer vision tasks and to establish a CVII benchmark for the COCO test set. Specifically, the CVII employs three hyperparameters, $\lambda_{1}$, $\lambda_{2}$, and $\lambda_{3}$, to calculate the CVII. To evaluate the significance of the hyperparameters, the experimental outcomes were systematically classified into five distinct groups denoted as CVII1, CVII2, CVII3, CVII4, and CVII5. These groupings are characterized by specific hyperparameter combinations, namely CVII1 ($\lambda_{1}=1$, $\lambda_{2}=1$, $\lambda_{3}=1$), CVII2 ($\lambda_{1}=1$, $\lambda_{2}=2$, $\lambda_{3}=3$), CVII3 ($\lambda_{1}=3$, $\lambda_{2}=2$, $\lambda_{3}=1$), CVII4 ($\lambda_{1}=1$, $\lambda_{2}=3$, $\lambda_{3}=2$), and CVII5 ($\lambda_{1}=2$, $\lambda_{2}=3$, $\lambda_{3}=1$). Considering these configurations, CVII4 is recommended for evaluating classification models, due to its emphasis on the classification ratio term. CVII3 is recommended for object detection models, as it assigns greater importance to the object detection ratio. CVII2 is suitable for segmentation models, given its heightened sensitivity to the correctness and mapping of the anchor box in alignment with segmentation. Finally, CVII1 is deemed appropriate for models combining all of the three primary tasks, as it weighs all of them equally, as discussed in Section 3.4.

The results, presented in Table 1 (first row), reveal a distinct correlation between the challenges associated with object identification in an image— encompassing object detection, segmentation, and classification— and the corresponding CVII scores. The average CVII values for the five hyperparameter combinations on all images (4975 images) in the COCO test set are presented in the first row of Table 1. These results were computed based on the annotations provided in the COCO dataset. Given that CVII3 exhibited the highest average among the different CVIIs, this suggests that, on average, the images in this dataset have superior object detection annotations. To a human annotator, the objects in these images were clearly discernible, and their anchors were relatively accurate.

Conversely, as CVII2 had a lower average value and emphasizes segmentation, these results indicated that the segmentation annotations were not as accurate. Various factors, such as crowded images, occluded objects, overlapping objects, or low resolution, may have contributed to this discrepancy. We explored this correlation using selected images, as showcased in the parameter evaluation section (Section 3.4). The findings strongly support a correlation between our claims and the CVII results.

In conclusion, our proposed methodology offers an efficient and effective approach for validating the interpretability of computer vision models on the COCO test set. It enables model developers to assess their models’ interpretability and abstractly compare their performance against an established benchmark.

### 3.3. CVII in a Real-World Setting

Our default pipeline for the baseline CVII incorporates three computer vision models: R-CNN Mask ResNet50 FPN for instance segmentation, YOLOv5 for object detection, and ResNet50 for image classification. These models capture the content and features of input images.

- R-CNN Mask ResNet50 FPN is an advanced instance segmentation model that accurately detects objects in an input image. It generates a list of detected objects with confidence scores and instance segmentation masks. By integrating Mask R-CNN ResNet50 FPN’s segmentation output with our architecture, we gain valuable insights into the image’s content and improve interpretability;
- YOLOv5 is a state-of-the-art object detection model that extracts meaningful features from input images. It uses a deep neural network architecture and multiple backbone networks. By incorporating YOLOv5 into our system, we can identify specific objects and their contribution to the CVII;
- ResNet50, a well-known deep neural network architecture, has been widely used for image classification and object detection tasks. To fine-tune the ResNet50 model, the 80 object types in the COCO dataset were organized into higher-order categories like animals, vehicles, and indoor items, depicting diverse aspects of daily life, we trained it using cropped images and employed transfer learning with pretrained weights. This process leveraged the model’s vast parameter space to improve its performance on a specific dataset.

In summary, the integration of R-CNN Mask ResNet50 FPN, YOLOv5, and ResNet50 into the CVII allowed us to gain valuable insights into the image content, improve interpretability, and enhance the overall performance of our system. The outcomes are presented in Table 1 (the second row). Compared to human-level interpretability based on the annotations provided in the COCO test set, the results indicate that the interpretability of the combined three-machine learning model approach was, on average, 27% lower than that of typical human decision-making on the same task, in other words, the CVII calculated on the basis of the human-supplied annotations of the COCO dataset (Table 1, first row) was higher by 27% on average compared to the CVII calculated for the given combination of ML models, reflecting the fact that human judgments are superior to machine generated outcomes in this case. The outcomes derived from deploying ML models in real-world scenarios align with our earlier findings based on the annotations of the COCO dataset. In both cases, CVII3 consistently exhibited the highest average score, while CVII2 consistently showed a lower value. This consistency validated that, on average, the segmentation task for the COCO dataset is comparatively easier than segmentation itself. This characteristic is reflected in our CVII scores across both settings.

Additionally, in both scenarios and comprehensively, CVII5 attained the second-highest value. This observation suggests that leveraging different models for interpretation enhances our overall interpretative capabilities. This provides evidence of a notable correlation between the complexity of object recognition in an image and the corresponding CVII. These findings support the validity of our approach, and underscore the need for further advancements in developing more interpretable computer vision systems, to enhance their reliability and trustworthiness.

### 3.4. Parameter Evaluation

This section investigates the impact of different hyperparameter combinations ($\lambda_{1}$, $\lambda_{2}$, and $\lambda_{3}$) on CVII and examines their significance by calculating CVII for five different combinations of these hyperparameters. The findings underscore the importance of customizing hyperparameters to suit specific requirements of the computer vision tasks.

To illustrate the influence of these hyperparameters, we selected five images from the COCO test set, each varying in difficulty for humans to identify all objects. We recorded the CVII values (CVII1–CVII5) for each image, as presented in Table 2. Notably, there was a consistent relationship between the degree of difficulty of each image and its computed CVII values. However, given that each CVII focuses on different aspects of the computer vision task, their correlations varied.

**Figure 4:**
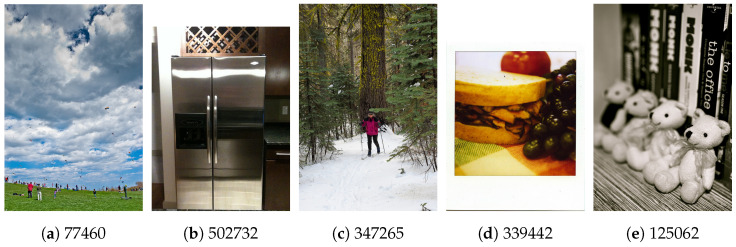
Five image instances in the COCO test set. Each has different characteristics based on their level of complexity, to detect each object in the images and distinguish them from their surrounding objects.

For instance, CVII2 places a higher emphasis on the accuracy of mapping object bounding boxes to segmentation contours, particularly with respect to $\lambda_{3}$. Consequently, when an image is densely populated or when precise boundaries for objects and bounding boxes are challenging to determine (e.g., in cases of overlap), CVII2 would typically yield the lowest value among the various CVIIs, as illustrated in Figure 4a.

On the other hand, $\lambda_{2}$ accentuates the importance of correct object classification. While our experiments relied on COCO dataset annotations with 100% classification confidence, we introduced a resolution factor based on the cropped object’s bounding box. This factor accounted for lower-resolution objects, which may lead to incorrect object classification. Furthermore, the choice of $\lambda_{2}$ is critical here. Table 2 demonstrates the CVII results for different images with varying object sizes and classification task difficulties.

Moreover, the $\lambda_{1}$ hyperparameter influences the detection rate factor. Increasing $\lambda_{1}$ amplifies the penalty imposed by CVII for lower detection rates. Thus, when an image is crowded, making it challenging for human observers to identify objects, or when inconsistencies exist between the number or type of objects detected by object detection and semantic segmentation models, a higher $\lambda_{1}$ value results in a greater CVII penalty. Conversely, higher $\lambda_{1}$ values emphasize this aspect and lead to a higher CVII when an image contains only a few large objects that are easily identifiable by both humans and models. This effect is evident in Table 2, where CVII3 achieved the highest value for images with fewer and larger objects, as expected (Figure 4b,d).

In addition, further analysis of Table 1 reveals variations in the interpretability of images within the COCO test set across different hyperparameter settings. Notably, CVII3 exhibited the highest interpretability score. This outcome can be attributed to the COCO dataset’s annotations, which generally include both bounding boxes and segmentation masks for detected objects. In contrast, CVII2, which emphasizes the match between bounding boxes and segmentation contours, yielded the lowest score. This reflects the challenges posed by overlapping objects and occlusions. CVII4, which factors in object resolution during classification, achieved a reasonable score, reflecting its consideration of resolution’s impact on interpretability.

The hyperparameters, require tuning based on the specific task at hand. For users employing the same platform and models, the suggested hyperparameter combinations from this paper can be directly applied. For instance, if classification takes precedence, opt for CVII4; for object detection, opt for CVII3; for semantic segmentation, opt for CVII2, and to balance interpretability across all tasks, opt for CVII1. Conversely, if distinct ML models are employed for each of the three main computer vision tasks, users should tune hyperparameters with various options to identify the optimal combination that best captures the desired interpretability feature. In general, for an emphasis on classification, assign greater weight to $\lambda_{2}$; for emphasis on object detection, assign more weight to $\lambda_{1}$, and for emphasis on segmentation, assign more weight to $\lambda_{3}$.

### 3.5. Case Study

#### 3.5.1. Case Study A

**Figure 5:**
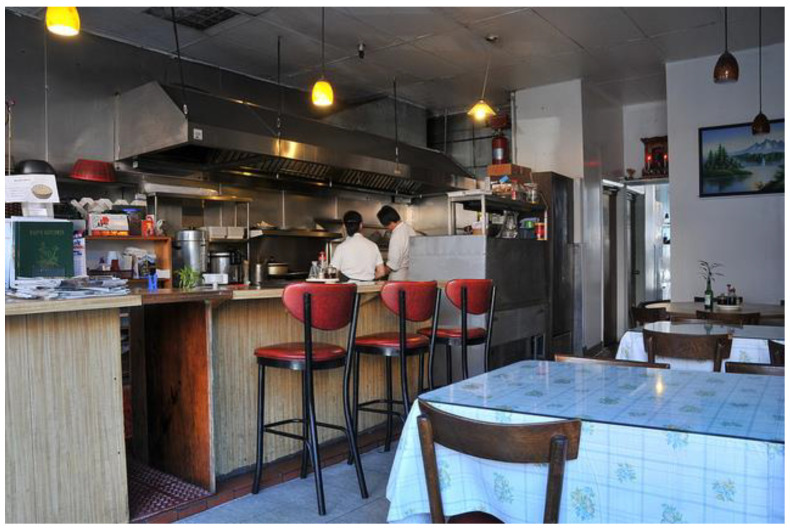
An image example used for the case study that explains the rationale behind CVII.

To further understand the rationale behind CVII and validate its efficacy, we showcase the correlation between CVII score and the interpretability of computer vision models. Given the image in Figure 5, according to the object detection model, there are 16 objects in this image, which is different from the object number derived from segmentation model that outputs 23 objects. Between them, there are five common objects, including ‘refrigerator’, ‘person’, ‘chair’, ‘dining table’, and ‘potted plant’, while missing five objects, including ‘bowl’, ‘cup’, ‘vase’, ‘traffic light’, and ‘bottle’. Among the objects common to both models, the object detection model detects 1 refrigerator, 2 persons, 9 chairs, 1 dining table, and 1 potted plant; in contrast, segmentation model detects 2 refrigerators, 2 persons, 8 chairs, 4 dining tables, and 2 potted plants. Also, among objects missed, object detection model detects 0 bowl, 0 cup, 0 vase, 0 traffic light, and 2 bottles, while segmentation model detects 2 bowls, 1 cup, 1 vase, 1 traffic light, and 0 bottles. Note that the ratio of the combined area covered by all objects in the image to the total size of the image is 0.3386 with the covered area as 92,087 pixels and image size as 272,000 pixels. Based on these observations, we can calculate the different CVII components about this image as follows:

(8) AF=0.0742,AC=0.0089,ASU=0.7089,IoU_AS=0.1573,CR=0.4478

Based on these component values, the CVII scores with different hyperparameter settings are as follows:

(9) CVII1=0.4930,CVII2=0.3731,CVII3=0.5650,CVII4=0.4311,CVII5=0.5155

These scores show inconsistencies between image segmentation and object detection. Addressing these disparities using our model involves cropping the areas of disagreement. Recomputation revealed that only one additional object detected by the segmentation model was deemed valid (i.e., a vase), while the others were deemed erroneous.

The calculated CVII scores corroborate this observation, with a higher value of CVII3, emphasizing the object detection task, compared to CVII2, which prioritizes the segmentation task. Additionally, examining CVII5, which assigns equal weight to all three tasks, yielded a relatively low score (0.5155). This outcome may be attributed to the image’s relatively low resolution and the presence of numerous and overlapping objects. This underscores the importance of operator discretion when making critical decisions based on images of this nature.

#### 3.5.2. Case Study B

In this case study, our objective was to survey a specific area using a camera-equipped sensory drone to identify zebras. The subsequent task involved immunizing the zebras by launching vaccine missiles. A drone captured images of the field, which we processed to generate a CVII score along with an explanation of how the model arrived at this score. The drone operator can leverage this interpretable information to make informed decisions regarding zebra identification and vaccination efforts. The CVII’s insights enhance the operator’s confidence, enabling effective zebra counting and vaccination procedures guided by the model’s interpretability.

**Figure 6:**
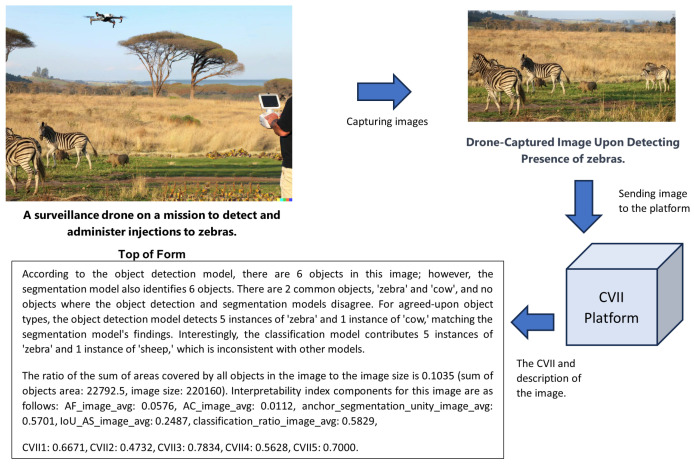
Scenario B: Utilizing the CVII platform for a zebra detection and immunization mission using a camera-equipped sensory drone.

As shown in Figure 6, CVII3 attained a higher score, due to accurate detection of most objects. Notably, misclassified objects like ’pig’ are not within the scope of the model’s training object types. Conversely, CVII2 achieved the lowest score, attributed to overlapping objects (’zebras’) and small object instances (’pigs’). Consequently, the model proved highly interpretable for tasks focused solely on object detection, instilling confidence in endeavors like zebra counting via drone imagery. However, if semantic segmentation takes precedence, reliance on the provided image may be precarious. For instance, deploying drone-delivered injection missiles based on this image would be unreliable. Note that ’pig’ is not included in the COCO dataset’s object types.

## 4. Challenges and Solutions

While calculating the CVII, we encountered several challenges. A significant hurdle was mapping objects between our semantic segmentation and object detection models. This task proved particularly challenging when dealing with models that generated different sets of objects and object types. To address this issue, we explored calculating the center of each segmented object and comparing it to the bounding boxes of the objects detected by the object detection model. However, a segmented object could be mapped to more than one object in the object detection model. To resolve this, we compared the confidence scores of each candidate mapping and selected the object with the highest confidence score. Furthermore, when the segmentation model detected more objects than detected by the object detection model, we either retained all objects in the object detection model or selected the best mapping and discarded the others, to avoid one object being mapped to multiple objects during the reverse evaluation. Ultimately, we found that using the intersection over union (IOU) to determine the mapping between objects in the two models was an effective approach that worked well for our dataset.

Another challenge we encountered when calculating the classification rate was accommodating the accuracy and resolution of our classification models. While higher resolutions typically produce more accurate results, most well-known models are trained on datasets with a resolution of 256 × 256 pixels. To address this, we developed a system for assigning confidence levels to our model outputs based on the size of the cropped objects being classified. For cropped objects with an area of 65,536 pixels, we assigned a confidence level of 1 to the model’s output, considering these as ideal inputs for classification models. For models trained on lower resolutions, such as 64 × 64 pixels, we estimated a lower confidence level of 0.9, to account for an expected 10% reduction in accuracy. We calculated a confidence rate within this range for cropped objects, with an area between 6536 and 4096 pixels, based on the area size of the cropped object, scaling down by 0.9 to account for the expected decrease in accuracy for models trained at lower resolutions. By implementing this approach, we effectively considered the impact of resolution and model accuracy on the classification of cropped objects.

## 5. Discussions on Limitations, and Ethical and Regulatory Compliance

### 5.1. Limitations

The limitations of our platform stem from the limitations of the underlying models and the choice of the datasets for training. Our work has some limitation stemming from the availability of the datasets used to evaluate different computer vision tasks. For instance, the choice of the COCO dataset for training/comparing computer vision models impacted the evaluation of object detection, semantic segmentation, and classification. The consistency of the dataset in terms of image sizes, the diversity of object types, the contextual breadth, and the accuracy of annotations was significant. Moreover, the robustness and capabilities of the deployed model such as the architectural design, hyperparameters governing the model’s behavior, and the delicate balance between bias and variance post-training significantly influenced the overall performance.

### 5.2. Ethical and Regulatory Compliance

The CVII framework has significant implications for ethical and regulatory compliance in AI-driven decision-making, contributing to transparency and accountability in several key ways:

- **Explainability and Trustworthiness:** By providing a quantitative measure of interpretability, the CVII enhances the explainability of AI models. Transparent models are crucial for establishing trust in AI systems, especially in decision-making processes that impact individuals and society at large. Stakeholders can better understand how models arrive at decisions, fostering trust and acceptance;
- **Ethical AI Development:** The CVII can serve as a guiding metric for ethical AI development. The framework encourages developers to consider interpretability during model design and training. This emphasis on interpretability aligns with ethical AI principles, ensuring that models are not “black boxes” and that their decisions can be scrutinized for fairness, bias, and adherence to ethical standards;
- **Regulatory Compliance:** As AI continues to evolve, regulatory bodies are actively seeking ways to ensure responsible AI deployment. The CVII offers a standardized metric that aligns with regulatory efforts. It provides a measurable benchmark for evaluating the interpretability of AI systems, aiding compliance with emerging regulations that mandate transparency and accountability in AI applications.
- **Human–AI Collaboration:** The CVII facilitates collaboration between humans and AI systems. In scenarios where AI-driven decisions impact human lives, having interpretable models allows human stakeholders to intervene, question, or provide feedback. This collaborative approach aligns with ethical considerations, recognizing the importance of human oversight in critical decision-making processes.

In conclusion, the CVII framework emerges as a valuable tool, not only for assessing the interpretability of AI models, but also for fostering ethical practices, regulatory compliance, and transparency in the rapidly advancing field of AI-driven decision-making. As society increasingly relies on AI technologies, the adoption of frameworks like CVII becomes crucial for ensuring responsible and accountable AI deployment.

## 6. Conclusions

In this paper, we introduced CVII, an interpretability index tailored for computer vision tasks, encompassing classification, object detection, and semantic segmentation, and also demonstrated its adequacy and versatility on the COCO dataset. Future work will include refining the model with knowledge distillation and extending the index to other machine learning domains, such as natural language processing, to enhance decision support in critical applications.

## Figures and Tables

**Table 1:** Comparison of the CVII calculated using COCO test set annotations vs. using three component computer vision models.

Using	CVII1	CVII2	CVII3	CVII4	CVII5
COCO Ann.	0.73898	0.58142	0.83352	0.66860	0.77721
ML models	0.53411	0.43121	0.59584	0.52809	0.59103

The employed models consisted of the Mask R-CNN ResNet50 FPN model for semantic segmentation, YOLOv5 for object detection, and a fine-tuned ResNet50 for classification. The reported numbers represent the average CVII score calculated across five distinct combinations of hyperparameters ($\lambda_{1}-\lambda_{3}$). The evaluation was performed on the entire COCO test set, utilizing the COCO dataset annotations. Additionally, the assessment was repeated on the same test set using our platform and employing the three aforementioned machine learning models.

**Table 2:** CVII for five image instances in the COCO test set. Each of them has different characteristics to better capture, compare, and enhance the nuances of the object detection, image segmentation, and classification.

Image No.	CVII1	CVII2	CVII3	CVII4	CVII5	Object Count	Object Types	Is Crowded
125062	0.89568	0.80905	0.94767	0.88243	0.93706	10	2	No
347265	0.58012	0.27791	0.76144	0.49187	0.69084	2	2	No
339442	0.97078	0.94958	0.98349	0.96476	0.97868	3	3	No
502732	0.99870	0.99740	0.99948	0.99870	0.99948	1	1	No
77460	0.29223	0.00510	0.52464	0.05192	0.33239	28	2	Yes

## Data Availability

The Microsoft COCO dataset [30] used in this study is publicly available, and can be downloaded from https://cocodataset.org/, 9 December 2023.
